# Improving symptom management for survivors of young adult cancer: rationale and study protocol for a pilot randomized controlled trial

**DOI:** 10.1186/s40814-024-01510-7

**Published:** 2024-06-08

**Authors:** Caroline S. Dorfman, Rebecca A. Shelby, Juliann M. Stalls, Samantha M. Thomas, Nicole A. Arrato, Brianna Herold, Tamara J. Somers, Francis J. Keefe, Joseph G. Winger, Jennifer Plumb Vilardaga, Kevin Oeffinger

**Affiliations:** 1 Department of Psychiatry and Behavioral Sciences, Duke University School of Medicine, Durham, NC USA; 2 Supportive Care and Survivorship Center, Duke Cancer Institute, Durham, NC USA; 3 Duke Cancer Institute, Durham, NC USA; 4 Department of Biostatistics and Bioinformatics, Duke University School of Medicine, Durham, NC USA; 5 Department of Medicine, Division of Medical Oncology, Duke University School of Medicine, Durham, NC USA

**Keywords:** Symptom management, Young adult, Oncology, Feasibility, Acceptability, Pilot randomized trial

## Abstract

**Background:**

Young adult (YA) cancer survivors are a growing, yet underserved population who often face significant and long-lasting cancer-related physical (e.g., pain, fatigue) and emotional (e.g., psychological distress) symptoms. Post-treatment symptoms can persist, disrupting YA’s abilities to complete goals consistent with their developmental stage (e.g., completing their education, achieving autonomy and independence, building their careers, establishing peer and romantic relationships, building their families). While symptom management has been identified as a significant issue in YA’s transitions to survivorship, the symptom management needs of this population largely go unmet.

**Methods:**

We developed an eight-session, group-based behavioral intervention that is delivered using videoconferencing to address the unique symptom management needs of YA cancer survivors. The intervention was developed in conjunction with YA survivors, leading to the novel combination of traditional behavioral symptom coping strategies, home-based physical activity, strategies from contemporary cognitive-behavioral approaches (e.g., those derived from acceptance and commitment therapy, strategies to foster self-compassion), concepts from meaning centered psychotherapy, and behavioral strategies to improve communication and health care engagement. Participants receive printed intervention materials and access to a study-specific mobile application, both of which are used throughout the program. Herein, we report on a pilot study that is in progress. Recruitment has been completed. YA cancer survivors were recruited in cohorts of *n* = 10 or *n* = 11 (*n* = 61) and randomized to the intervention or waitlist control arms. All participants completed a baseline assessment and four additional assessments over 1 year, with each involving a battery of self-report measures.

**Discussion:**

The primary objective is to evaluate intervention feasibility and acceptability. As a secondary objective, we will examine patterns of change in intervention targets (i.e., pain, fatigue, emotional distress, symptom interference). Changes from baseline among intervention targets will be estimated for each patient and compared between arms using unadjusted statistical testing. Unadjusted and adjusted multilevel modeling will be used to estimate the effect of the intervention on changes in intervention targets. Interaction models will be used to compare the trajectory of change over time between arms. We expect that this pilot trial will inform our future approach to identify, recruit, and retain participants and provide preliminary data to support a larger, fully powered randomized controlled trial evaluating the intervention.

**Trial registration:**

NCT04035447 at clinicaltrials.gov; registered July 29, 2019.

**Supplementary Information:**

The online version contains supplementary material available at 10.1186/s40814-024-01510-7.

## Background

In the USA, there are more than 600,000 survivors of young adult (YA) cancer, aged 18–39 at diagnosis [1–3]. This population will continue to grow over the next decade, with more than 85% surviving at least 5 years [1]. For many YA cancer survivors, the impact of treatment is significant and long-lasting, including difficult physical symptoms (e.g., pain, fatigue) [4–6], psychological distress [6–10], and an increased risk of long-term health problems (e.g., second cancers, early-onset cardiovascular disease) [11–13].

For YA cancer survivors, persistent post-treatment physical symptoms and emotional distress occur during a critical developmental period and can disrupt YA’s abilities to complete their education, achieve autonomy and independence, build their careers, establish peer and romantic relationships, and build their families [14]. YA report significant unmet symptom management needs [4, 15, 16], and when compared to older or younger cancer survivors, YA report more difficulties and less confidence in their abilities to manage physical and emotional symptoms [17]. Consequently, physical symptoms and emotional distress contribute to significant social, economic, and health burdens for YA at a time when they should otherwise be on an upward social, economic, and health trajectory [18–22]. While symptom management has been identified as a significant issue in YA’s transitions to survivorship [23, 24], YA’s symptom management needs largely go unmet.

The National Cancer Policy Forum and National Cancer Institute have highlighted the critical need to assist patients with reducing symptom burden and promoting self-management of symptoms [12, 13]. Yet, there remains a paucity of evidence-based interventions to improve the symptom burden and optimize the long-term healthcare of YA survivors. While YA survivors may be prescribed medications to help manage symptoms [25], this approach is often insufficient, can result in new and/or additional side effects [26], and fails to address the psychological and behavioral factors that impact and exacerbate symptom burden in this population. Existing behavioral symptom management interventions for cancer survivors have been primarily developed and tested among older cancer survivors (aged > 40) [27–32]. Thus, the applicability of these interventions to the experiences of YA cancer survivors is unknown.

To begin to fill this gap, we recently developed an 8-session behavioral symptom management intervention to improve YA survivors’ abilities to better manage physical symptoms (i.e., pain, fatigue) and emotional distress [33]. Throughout the intervention development phase, input was obtained from YA cancer survivors and oncology clinicians to ensure that content and examples were relevant to the unique experiences of this population. The developed group-based intervention is delivered using videoconferencing and includes the novel combination of the following: (1) traditional behavioral symptom coping strategies (e.g., relaxation training, activity pacing); (2) home-based physical activity (i.e., cardiovascular, strength, and resistance training); (3) strategies from contemporary cognitive-behavioral approaches (e.g., acceptance and commitment therapy (ACT)-based strategies [34], like cognitive defusion and movement towards valued action; strategies to foster self-compassion, like loving kindness meditations [35–37]); (4) concepts from meaning-centered psychotherapy (MCP; e.g., building meaning, focusing on the legacy you live and give) [38–41]; and (5) strategies to improve communication and health care engagement (e.g., assertiveness training). Participants are provided with printed intervention materials and access to a study-specific mobile application, both of which are used throughout their participation in the program. The goal of the intervention is to assist YA cancer survivors with better coping with cancer and treatment-related symptoms in the service of engaging in valued and meaningful activities congruent with their developmental stage.

## Study aims

The primary objective of the pilot randomized controlled trial is to evaluate the feasibility of study participation and the acceptability of the developed behavioral symptom management intervention for YA cancer survivors. Intervention feasibility will be assessed through the session attendance rate, assessment completion rate, attrition rate, and participants’ use of intervention strategies. Intervention acceptability will be examined through participants’ self-reported assessments of treatment acceptability and satisfaction with the intervention.

Secondary study aims are to examine patterns of change in intervention targets including physical (i.e., pain, fatigue) and emotional (i.e., symptoms of depression and anxiety) symptom severity as well as change in symptom interference for participants in the intervention and waitlist control arms. As an exploratory aim, change in self-efficacy for symptom management and social-support from pre- to post-treatment will be examined as mediators of group differences in symptom severity and interference.

**Fig. 1:**
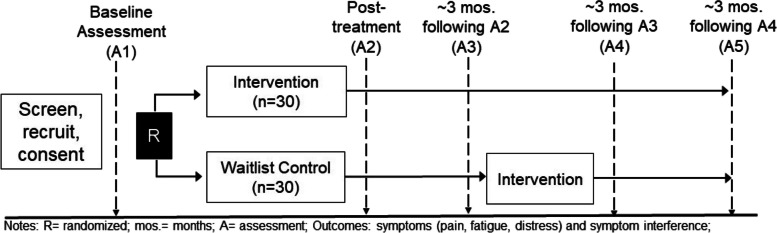
Study design. RCT with *n* = 60 survivors of young adult cancer

This protocol manuscript was prepared in accordance with the 2013 Standard Protocol Items: Recommendations for Intervention Trials (SPIRIT) guidelines. A SPIRIT figure outlining the schedule of enrollment, interventions, and assessment is presented in Fig. 1. The SPIRIT checklist has been included as an Additional File.

## Methods

### Participant eligibility criteria

Participants were recruited through a National Cancer Institute-designated comprehensive cancer center in the southeast of the USA. Eligible participants had a diagnosis of hematologic, breast, endocrine (e.g., thyroid), or gastrointestinal cancer, melanoma, or germ cell tumor (e.g., testicular, ovarian) and were diagnosed with cancer between the ages of 18 and 39 [1]. These cancer types were selected because they represent six of the most common cancers among YA in the USA. Additional eligibility criteria include the following: (1) aged 18–39 at enrollment; (2) completed curative treatment involving multimodal therapy (i.e., at least two types of treatment) within the last 2 years; (3) able to speak/read English; and (4) able to provide informed consent. Exclusion criteria included the following: (1) non-ambulatory; (2) major mental illness (e.g., schizophrenia); and (3) untreated or uncontrolled mental illness (e.g., bipolar disorder). Confirmation was obtained from a member of participants’ medical team that the participant was able to engage in home-based exercise.

### Participant recruitment

This NIH-funded study (K08CA245107) received Institutional Review Board (IRB) Approval (Pro00103249, most recent amendment February 23, 2023; NCT04035447, registered July 29, 2019, last updated July 26, 2023). Potentially eligible participants were identified through as follows: (a) electronic health records of the larger health system; and (b) from an ongoing, IRB-approved (Pro00100124) observational study that included YA cancer survivors treated at our institution who had consented to being re-contacted for future research studies. Information about potential participants (e.g., name, date of birth) is stored in a password-protected database, for recruitment and tracking purposes. Participants were provided information about the study through a letter and study brochure mailed to their homes or sent via the health system’s electronic health record. Participants were then contacted via telephone by study staff, who provided additional information about the study procedures and confirmed participant eligibility. In order to reduce the duration of in-person contacts, participants were given the option to complete the informed consent process at home online via Research Electronic Data Capture (REDCap) [42, 43] or in-person, both with the support of trained study staff.

### Trial design and randomization

The study involves a pilot randomized controlled trial, which was designed and will be reported in accordance with CONSORT guidelines [44]. Sixty-one YA cancer survivors were recruited for participation, in six cohorts. Figure 1 presents the study design and timeline. Following informed consent, participants were asked to complete a set of questionnaires online using REDCap. At the baseline assessment (A1), participants were asked to provide information about their sociodemographic characteristics as well as information about their physical health and cancer history. Participants also completed self-report measures (see below) associated with intervention targets (i.e., pain, fatigue, emotional distress, symptom interference, physical activity, values, spirituality) and potential intervention mechanisms (i.e., self-efficacy for symptom management, social support).

Next, participants were randomized within their cohort to either the intervention arm or to the waitlist control arm in a 1:1 ratio. Randomization was stratified by gender; however, after randomizing participants in cohort 1, it was recognized that the initial randomization scheme would not produce balanced groups for the intervention and control arms because it did not adequately account for differing sizes of stratified groups (i.e., disproportionately high number of female versus male participants) and did not incorporate a block design. The study biostatistician was consulted to develop a new randomization scheme for cohorts 2–6. The randomization scheme is implemented using the REDCap randomization module, and trial staff were blinded to the randomization scheme. A random permuted block design was used stratified by participant self-described gender (female vs. non-female). An estimated enrollment of 75% female to 25% non-female participants was used to inform block sizes of four for female participant and two for non-female participant randomization to ensure balance throughout enrollment. Once randomized, neither trial staff nor study participants were blinded to participants’ intervention arm status.

Following randomization, intervention arm participants receive the 8-session behavioral symptom management intervention, which is delivered over the course of ~ 10 weeks. Figure 1 outlines the timing of follow-up assessments. Upon completion of the study intervention, both intervention and control arm participants complete the second assessment (A2). Participants in both arms complete three additional assessments (A3–A5) approximately every 3 months, involving the same self-report measures as the baseline assessment. Participants in the waitlist arm initiate the behavioral symptom management intervention following completion of the third assessment (A3). Participants complete measures of intervention acceptability, satisfaction, and use of intervention strategies at A2 (intervention arm) or A4 (control arm).

### Study enrollment

Study recruitment began in August 2021 and was completed in May 2023. Due to patient interest, one additional individual was enrolled for a total sample size of *n* = 61 participants. Five of six cohorts included *n* = 10 YA, with the final cohort including *n* = 11 YA. Study intervention sessions and assessments are ongoing. Study sessions are expected to conclude in January 2024 with assessments concluding in April 2024.

Participants’ sociodemographic and medical characteristics are presented in Table 1. YA participating in the pilot randomized controlled trial were on average 32.4 years old, and the majority were White (*n* = 44, 72.1%). Just under three-fourths of the sample identified as female (*n* = 44, 72.1%). The majority of participants were working full or part time for pay (*n* = 39, 63.9%), were partnered (*n* = 38, 62.3%), and had health insurance (*n* = 60, 98.4%). Participants had been diagnosed with breast (*n* = 28), hematologic (*n* = 15), testicular (*n* = 7), endocrine (*n* = 7), or gastrointestinal (*n* = 3) cancers or melanoma (*n* = 1). 83.6% of patients received surgery, 80.3% received chemotherapy, and 50.8% received radiation.

**Table 1:** Sociodemographic and medical characteristics of young adult survivor participants (*n* = 61)

	*N* (%)	Mean (SD)/range
Age at study enrollment		32.36 (4.71)/22–39
Race		
African American	7 (11.5%)	
Asian	3 (4.9%)	
White	44 (72.1%)	
American Indian/Alaska Native	1 (1.6%)	
More than one race	4 (6.6%)	
Other	2 (3.3%)	
Ethnicity		
Hispanic	5 (8.2%)	
Not Hispanic	56 (91.8%)	
Gender		
Male	17 (27.9%)	
Female	44 (72.1%)	
Education		
Completed high school	3 (4.9%)	
Some college, vocational, or training school	7 (11.5%)	
Associate degree	6 (9.8%)	
College graduate	19 (31.1%)	
Some graduate work/graduate degree	26 (42.6%)	
Employment status		
Student	4 (6.6%)	
Working full or part-time for pay	39 (63.9%)	
Unemployed	4 (6.5%)	
Full-time homemaker or family caregiver	5 (8.2%)	
On disability	4 (6.6%)	
Other	2 (3.3%)	
Missing	3 (4.9%)	
Health insurance		
Yes	60 (98.4%)	
No	1 (3.3%)	
Type of health insurance (*n* = 60)*		
On parent’s insurance	5 (8.3%)	
Provided by an employer/former employer	38 (63.3%)	
Government-sponsored health insurance (e.g., Medicaid,	11 (18.0%)	
CHIP, Social Security Disability)		
On spouse’s/partner’s insurance	9 (15.0%)	
Relationship status		
Single (never married)	21 (34.4%)	
Married/partnered	38 (62.2%)	
Divorced	1 (1.6%)	
Separated	1 (1.6%)	
Responsible for raising children aged < 18		
Yes	26 (42.6%)	
No	35 (57.4%)	
Cancer type		
Breast	28 (45.9%)	
Endocrine (e.g., thyroid)	7 (11.5%)	
Melanoma	1 (1.6%)	
Gastrointestinal	3 (4.9%)	
Germ cell (i.e., testicular)	7 (11.5%)	
Hematologic	15 (24.5%)	
Treatments*		
Chemotherapy (*n* = 61)	49 (80.3%)	
Radiation (*n* = 59)	31 (50.8%)	
Hormonal treatment (*n* = 56)	20 (32.8%)	
Surgery (*n* = 58)	51 (83.6%)	
Radioactive iodine (*n* = 52)	7 (11.5%)	
Immunotherapy (*n* = 52)	10 (16.4%)	
Other^+^	8 (13.1%)	
Experienced a cancer recurrence	5 (8.2%)	

*Numbers do not add up to 100% if participants reported more than one response;

^+^Other treatments include the following: complementary and alternative medicines, hematopoietic stem cell transplant, anti-HER2 therapy, CDK4/6 inhibitors

## Study intervention

The eight-session group-based behavioral symptom management program is delivered using a faded contact approach (i.e., sessions 1–6 weekly, sessions 7–8 biweekly) to provide participants sufficient time to engage with skills and home-practice assignments. The sessions are approximately 90 min long and delivered remotely using videoconferencing technology (i.e., Zoom). Informed by social cognitive theory [45, 46], intervention sessions include opportunities for modeling, role play, and receiving feedback as well as opportunities for self-evaluation, self-monitoring, and goal setting. Each session follows the same structure: (1) group socialization (15 min); (2) review home practice and skill and mobile application use (20 min); (3) provide education, skills training, and opportunities for skills practice and applying skills to their own experiences (45 min); and (4) assign home practice (10 min).

Each session is facilitated by two study interventionists who are psychologists (i.e., one doctoral-level and one master’s-level psychologist). Sessions are structured to promote connection with other group members (e.g., open-ended questions to facilitate group discussion), enhance skills application, and build self-efficacy for symptom management. Participants are encouraged to be present on camera, ask questions of facilitators, and have informal and formal interactions with other participants to foster an environment most similar to an in-person session. In the event that a participant is unable to attend a scheduled group session, they are offered a make-up session delivered by one of the group facilitators so that they can more easily return to the next group session.

### Outline of sessions

#### Session 1

At the start of session 1, participants are provided with a program overview. The biopsychosocial-spiritual model [47, 48] is presented as a framework for considering the impacts of cancer on YA’s lives and used to frame the intervention strategies to be discussed later in the program. Participants are asked to share their cancer story, specifically noting cancer’s impacts on their lives and goals for the future as well as any symptoms they are managing post-treatment. Next, participants are introduced to the benefits of physical activity for the management of symptoms like pain, fatigue, and emotional distress. Study-specific tools (see below) are reviewed to support their engagement in home-based physical activity including a wireless activity tracker and physical activity manual. The physical activity manual provides a week-by-week home-based exercise program with content focusing on increasing engagement in aerobic activity (i.e., walking or walk-to-run-program), resistance training (e.g., body weight exercises), and flexibility exercises (e.g., dynamic and static stretches). The session concludes with psychoeducation about the benefits of relaxation for symptom management, and the participants are guided through a progressive muscle relaxation (PMR) practice.

#### Session 2

Participants are provided psychoeducation about values. They are asked to engage in a variety of experiential exercises to identify their personal values and value-driven goals [49]. They are then introduced to the S.M.A.R.T. (i.e., specific, measurable, achievable, relevant, time-oriented) goal framework and asked to set one or more value-driven S.M.A.R.T. goals. To support their progress towards value-driven S.M.A.R.T. goals, they are also taught about the benefit of activity pacing (i.e., activity-rest cycling) for symptom management. Participants are supported in using activity pacing to begin to work towards a value-congruent goal.

#### Session 3

Participants are provided psychoeducation about the role of thoughts in symptom management, common unhelpful thinking patterns, and the utility of noticing unhelpful thoughts. The “Monsters on the Bus” metaphor from Acceptance and Commitment Therapy [50] is employed to illustrate the ways in which “being hooked” by unhelpful thoughts may sometimes pull participants away from values-driven activities. Strategies for noticing when they are “hooked” by unhelpful thoughts (e.g., recognizing common thinking patterns, recognizing the emotional, physical, and behavioral consequences of unhelpful thoughts) are reviewed. Participants are encouraged to track their personal physical and emotional consequences of being “hooked.” Psychoeducation is also provided about the potential to be unkind to ourselves when we are “hooked” by unhelpful thoughts or when our thoughts move us away from values-driven activities. Strategies to foster self-compassion and loving-kindness are introduced. Participants are guided through an experiential practice of self-compassion (i.e., “How would you treat a friend?” exercise) as well as a formal loving-kindness meditation practice [35–37].

#### Session 4

Our discussion about the role of thoughts in symptom management is continued. Beyond the utility of noticing thoughts and their behavioral impacts discussed in session 3, participants are introduced to the concept of obtaining distance from their thoughts through the use of cognitive defusion strategies (e.g., labeling thoughts, “silly voices,” the leaves on a stream exercise). Participants are guided through experiential practice of cognitive defusion strategies [51, 52]. Participants are also provided with psychoeducation regarding workable or helpful self-talk statements. The leaves on a stream exercise are introduced as a defusion strategy that may also foster a sense of relaxation. Participants are guided through an in-session practice of the leaves on a stream exercise [52].

#### Session 5

Psychoeducation is provided about the value of support from close others for symptom management. Participants are guided through identifying members of their social support network, and are encouraged to match their support needs with individuals in their network who are best suited to provide support (i.e., task support vs. emotional support). Psychoeducation is provided about the value of assertive communication in contrast to passive or aggressive communication for getting support needs met and for saying no to requests from others. Participants are encouraged to make an individualized plan for utilizing assertive communication skills. Group discussion is facilitated around ways to manage distress that may occur when support needs are not met, even after making an effective support request. Many YA cancer survivors feel as though others in their life, including peers and family members, do not understand their experience as a young person with cancer [53, 54]. Participants are led through a group discussion of strategies to cope in these situations, and they are provided with information about YA-specific programming available at our institution and through national organizations to help connect with other YA survivors. Informal loving-kindness meditation is introduced as a strategy to cope when participants have not received needed or requested support.

#### Session 6

Our discussion of communication is continued, with a focus on communicating with medical providers and communicating at work or school. Barriers to communicating with medical providers about symptoms are discussed. Participants are encouraged to identify questions to ask their providers prior to their appointments, and assertive communication is reviewed as a strategy to foster effective communication with the medical team. Next, participants’ disclosure of their cancer diagnosis and symptoms at work and/or at school are discussed, and assertive communication is again reviewed as a strategy to facilitate cancer-related conversations in these contexts. Participants are provided with resources (e.g., national organizations concerned with assisting cancer survivors at work and in school) to help facilitate cancer-related conversations. An overview of laws designed to protect cancer survivors in the workplace and at school is provided, and participants with additional questions in this area are encouraged to seek legal support. Participants are guided through an experiential mini-relaxation practice (i.e., brief relaxation practice combining paced breathing and muscle relaxation) to assist with coping during challenging interactions with others.

Following session 6, intervention sessions move from weekly to biweekly. A review is provided of the skills discussed in the program thus far, and participants are supported in developing a plan for continuing to use program skills in the time between sessions.

#### Session 7

Psychoeducation is provided about meaning and meaning-making following cancer. Participants are guided through exercises to identify sources of meaning in their lives, reflect on their identity before and after cancer, and to understand the impact that cancer may have had on their sense of meaning [40]. Participants are asked to identify strategies to cope with change or loss of meaning following cancer and to identify activities that are personally meaningful (e.g., spending time in nature). A guided imagery practice (i.e., visualization of a peaceful scene that is personally meaningful) is introduced as a skill to foster a sense of meaning.

#### Session 8

During the final session, we conclude our discussion of meaning by helping participants identify the legacy that they hope to live now as they build their life story and give to others. Participants are guided through exercises to help them: (1) understand the significant values, traditions, and memories that have impacted who they are today; (2) reflect on their meaningful activities, roles, and accomplishments; and (3) make intentional choices to live in a way that is consistent with their values and the legacy they hope to live and give [40]. Participants are assisted with reviewing and celebrating their individual progress in the program, and are supported in making a plan for continued practice of program skills. Participants are given information about support services and resources available both within and outside of our institution should they be interested in additional support following program completion. They are reminded about next steps in the program (i.e., completion of study assessments) and are encouraged to utilize the study team as a resource to help them to connect with additional support services, as needed.

### Intervention tools

Participants are provided with four tools to support their engagement in the behavioral symptom management program. (1) Participants receive a printed intervention manual that provides a session-by-session program outline, written descriptions of coping skills taught in the context of the program, and home practice assignments. (2) Participants receive a printed home-based physical activity manual, which provides physical activity education along with a structured walking program, walk-to-run program, strength training program, and flexibility program. This manual was developed in collaboration with exercise physiologists who specialize in working with cancer patients. (3) Participants receive a Garmin Vivofit 4 wireless activity tracker to increase their awareness of their personal daily activity level. The Garmin Vivofit 4 was selected due to its ability to synchronize with the study-specific mobile application and long battery life (i.e., 1 year). (4) Participants receive access to a study-specific mobile application developed by Pattern Health. The mobile application is accessible on both Android and iOS devices. Participants are assisted with downloading the mobile application and setting up the activity tracker prior to the start of study sessions.

The mobile application provides participants with on-the-go access to intervention strategies (e.g., recorded audio files of relaxation and loving kindness meditations, videos the movements used in the resistance and flexibility programs, coping strategy descriptions), tools to support their uses of coping strategies (e.g., Spotify playlists to use while engaging in physical activity, space to enter information about home-practice assignments; see Table 2), and the ability to chat with other group members through a secure, moderated chat platform. The chat is moderated by the study interventionists, who receive email notifications when a group member submits a message to the chat.

**Table 2:**
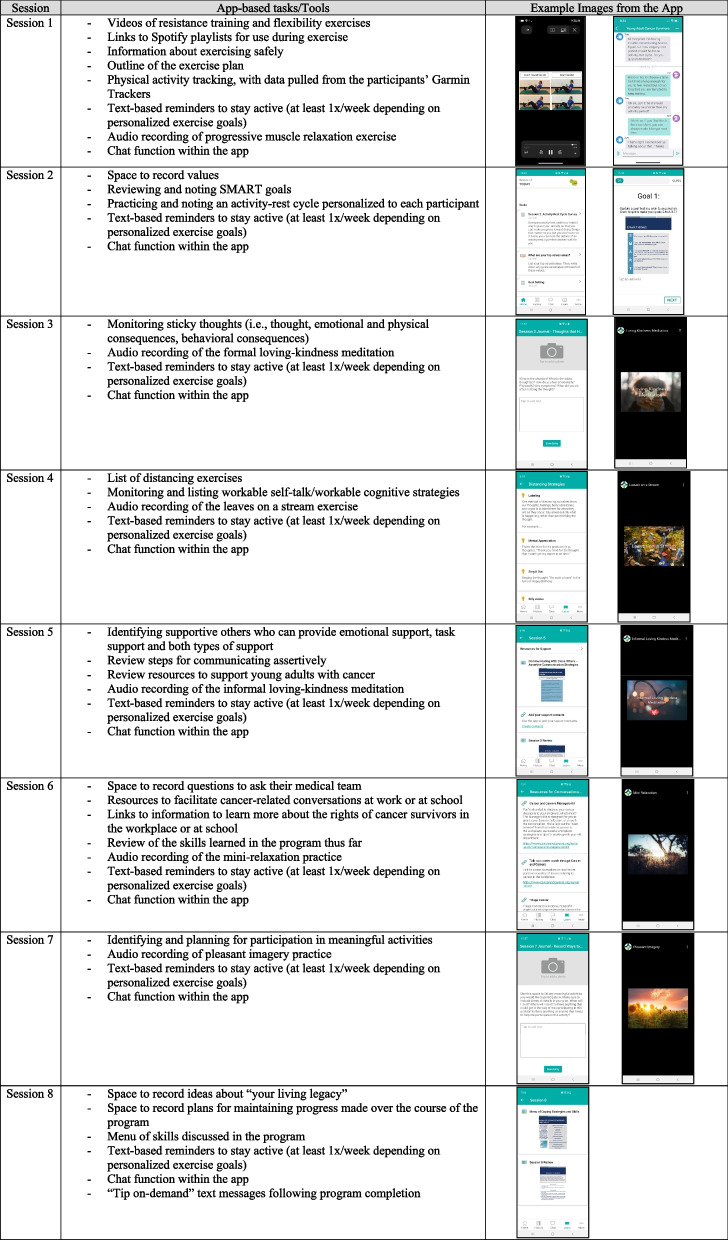
Session-by-session outline of home practice assignments within the mobile application (app)

The mobile application also provides regular, personalized messages through push notifications and text messages that serve as reminders to practice study-specific coping skills (i.e., weekly) and work towards/modify study-specific goals (e.g., every 3 days). Participants are prompted by the application to complete periodic symptom check-ins, during which they are asked to provide symptom ratings and can receive a symptom coping tip if interested in further assistance with navigating a particular symptom. Participants can earn badges for completing study-specific home practice activities (i.e., completing half of the week’s content). The mobile application tracks a variety of components of participants’ engagement with the tool, including the frequency of logins, the number of completed assignments, and the frequency with which they access audio and video recordings. Participants are encouraged to continue to use the application after completing study sessions and have access to the mobile application content for up to 1 year following intervention completion.

### Waitlist control arm

Participants randomized to the waitlist control arm receive usual medical follow-up care and initiate intervention sessions after all participants within their cohort complete the third assessment (see Fig. 1).

### Participant engagement newsletters

To facilitate participant engagement in the study in the time between study assessments, all participants are mailed brief newsletters. The first newsletter is sent approximately 6 weeks following the intervention arm’s initiation of the symptom management program. Subsequent newsletters are sent approximately 6 weeks following assessments two, three, and four. Newsletters cover topics of relevance to YA cancer survivors that are distinct from content covered in the symptom management program, including (1) nutrition; (2) sleep; (3) finances; and (4) cognitive problems. Each newsletter briefly outlines one topic, provides education about the value of the topic for YA survivors, offers tips for addressing the topic, and introduces resources for obtaining more information about the topic.

## Interventionist training and fidelity

To ensure consistency of intervention delivery, study therapists received didactic instruction for each intervention session by the study principal investigator (CSD). A written therapist manual was also created to support fidelity of intervention delivery. The therapist manual includes scripted text to use to deliver each of the intervention sessions. The therapist manual also includes question prompts to ask of the group along with tips about how to respond to participants’ comments and engage participants with the content. The intervention sessions are audio/video recorded. The recorded sessions were viewed by new therapists as a part of the intervention training and onboarding. Approximately 20% of the study sessions will be reviewed by a study team member (BH or NAA) using a fidelity checklist to assess fidelity of intervention delivery over the course of the study.

## Safety protocols

All participants continue to be monitored by their physicians throughout the course of the study; participants’ doctors provide monitoring of their overall medical status. All members of the study team who have direct contact with participants are trained to observe and report any adverse events. Adverse events are reported to the institution’s IRB in real time. We have also appointed two data and safety officers. One of the data and safety officers is a medical doctor, who is not affiliated with the study, and who has experience in clinical research and trials as well as a thorough understanding of adverse events. The second data and safety officer is a health psychologist with expertise in behavioral symptom management. The study team meets with the data and safety officers annually to review our current methods of assessment and intervention and identify any problems. In addition to reporting adverse events to the IRB, adverse events are reported to the data safety officers in real time.

## Study outcomes and measures

Each assessment (A1 through A4) includes self-report measures with extensive reliability and validity data. Table 3 outlines the timing of each measure by study arm. Participants complete assessments online using REDCap, and data is stored securely on the health systems servers behind a fire wall. Only members of the study team who are key personnel on the IRB application have access to study data. Participants are identified in study data using a unique study identification number.

**Table 3:** Study assessment components administered at each time point

	Baseline (A1)	A2	A3	A4	A5
**All participants**					
Sociodemographics	X				
Diagnosis and treatment information	X				
Brief Pain Inventory (9-item)	X	X	X	X	X
PROMIS Fatigue Scale (8-item)	X	X	X	X	X
PROMIS Depression Short Form (8-item)	X	X	X	X	X
PROMIS Anxiety Short Form (8-item)	X	X	X	X	X
Illness Intrusiveness Rating Scale (13-item)	X	X	X	X	X
Self-Efficacy for Managing Chronic Disease Scale (6-item)	X	X	X	X	X
PROMIS Self-efficacy for Managing Chronic Conditions Short Form (4-item)	X	X	X	X	X
PROMIS Emotional Support Short Form (6-item)	X	X	X	X	X
PROMIS Instrumental Support Short Form (6-item)	X	X	X	X	X
PROMIS Informational Support Short Form (6-item)	X	X	X	X	X
PROMIS Social Isolation Scale (6-item)	X	X	X	X	X
International Physical Activity Questionnaire (7-item)	X	X	X	X	X
Stanford LCAT (1-item)	X	X	X	X	X
Functional Assessment of Chronic Illness Therapy (FACIT)- Spiritual Well-being (12-item)	X	X	X	X	X
Acceptance and Action Questionnaire for Cancer Version 2 (7-item)	X	X	X	X	X
Valuing Questionnaire (10-item)	X	X	X	X	X
**Intervention arm**					
Treatment Acceptability Questionnaire (6-item)		X			
Satisfaction with Therapy and Therapist Scale-Revised (13-item)		X			
Open ended questions about the program		X			
Self-reported use of intervention skills		X	X	X	X
Self-reported use of mobile app		X	X	X	X
Group Therapy Experiences Scale (17-item)		X			
**Waitlist control arm**					
Treatment Acceptability Questionnaire (6-item)				X	
Satisfaction with Therapy and Therapist Scale-Revised (13-item)				X	
Open ended questions about the program				X	
Self-reported use of intervention skills				X	X
Self-reported use of mobile app				X	X
Group Therapy Experiences Scale (17-item)				X	

### Aim 1: treatment feasibility, acceptability, and satisfaction

#### Feasibility

Treatment feasibility is assessed by measuring the session attendance rate for each participant. Participant attrition will also be examined. Participants’ self-reported use of intervention skills and the mobile application will be examined along with responses to open-ended questions asking about their views of the intervention. The intervention will be deemed feasible if participants complete > 80% of scheduled intervention sessions and > 80% of study assessments.

#### Acceptability

The 6-item Treatment Acceptability Questionnaire (TAQ) [55] assesses participants’ views of the intervention as ethical, acceptable, and effective as well as their perceptions of the interventionists’ knowledge and trustworthiness. Items are rated on scales from 1 to 7, with lower scores indicating less of the construct assessed (e.g., 1 “very unacceptable” to 7 “very acceptable”; 1 “unethical” to 7 “very ethical”). The intervention will be deemed to be acceptable if > 80% of participants rate the intervention as an average score of 5 or greater on the TAQ.

#### Satisfaction

The 13-item Satisfaction with Therapy and Therapist Scale-Revised (STTS-R) [56] assesses participants’ satisfaction with the symptom management program and the interventionists delivering the program. The measure was modified slightly to reflect the intervention being delivered (e.g., “I am now able to more effectively deal with my symptoms”). We will examine mean scores on the STTS-R and the percent of participants rating domains associated with the program (e.g., therapy, therapist) as satisfactory.

### Aim 2: patterns of change in symptom severity and interference

#### Pain severity and interference

The Brief Pain Inventory [57] assesses both pain severity and interference. Pain severity is calculated as the average of four items assessing participants’ worst, least, current, and average (in the last week) pain on a scale from 0 “no pain” to 10 “worst pain.” Pain interference is computed as the average of seven items, which ask about the interference of pain across different life domains (e.g., general activity, mood, relations with other people).

#### Fatigue

The 8-item short form of the Patient Reported Outcomes Measurement Information System (PROMIS)-Fatigue Scale [58] assesses fatigue symptoms (e.g., I felt fatigued, how much were you bothered by your fatigue on average) in the last week with scales ranging from 1 to 5. Corresponding item responses vary from “not at all” to “very much” or “never” to “always” depending on the item. Items are summed and converted to standardized *t*-scores.

#### Depressive symptoms

The 8-item short form of the PROMIS-Depression Scale [58] assesses symptoms of depression (e.g., I felt hopeless, I felt worthless) in the last week on a scale from 1 “never” to 5 “always.” Items are summed and converted to standardized *t*-scores.

#### Symptoms of anxiety

The 8-item short form of the PROMIS-Anxiety Scale [58] assesses symptoms of anxiety (e.g., I felt nervous, my worries overwhelmed me, I felt anxious) in the last week on a scale from 1 “never” to 5 “always.” Items are summed and converted to standardized *t*-scores.

#### Symptom interference

The 13-item Illness Intrusiveness Rating Scale [59] assesses how much one’s illness and/or its treatments interfere with different life domains (e.g., health, work, finances, relationships with significant others) on a scale from 1 “not very much” to 7 “very much.” Items are summed, with scores ranging from 13 to 91; higher scores indicate greater symptom interference.

### Exploratory aim: patterns of change in self-efficacy and social support as mediators of group differences in symptom severity and interference

#### Self-efficacy for symptom management

The 6-item Self-Efficacy for Chronic Illness Scale [60, 61] assesses participants’ current confidence in their ability to prevent symptoms (e.g., pain, fatigue, distress) from interfering with the things they want to do. Items are averaged, with higher scores indicating greater self-efficacy. The 4-item PROMIS Self-Efficacy for Managing Chronic Conditions-Managing Symptoms Scale [62] assesses participants’ current confidence in their ability to manage symptoms in daily activities, in relationships, in public places, and in working with their doctors. Items are summed and converted to standardized *t*-scores.

#### Social support

Four PROMIS short form instruments [58] are used to assess different domains of social support: (1) the 6-item short form of the PROMIS Emotional Support Scale; (2) the 6-item short form of the PROMIS Instrumental Support Scale; (3) the 6-item short form of the PROMIS Informational Support Scale; and (4) the 6-item short form of the PROMIS Social Isolation Scale. Items are rated from 1 “never” to 5 “always.” Items on each instrument are summed and converted to standardized *t*-scores.

The 17-item Group Therapy Experiences Scale [63, 64] assesses the level of cohesion among group members during their participation in the program (e.g., development of positive relationships, comfort level with other group members). Items 1–16 are rated on a 5-point scale from 1 “strongly agree” to 5 “strongly disagree.” Item 17 is an open-ended question: “Was there something in the group today that helped or hindered you?”.

### Other intervention targets

#### Physical activity

The 7-item short form of the International Physical Activity Questionnaire [65] assesses the amount of time (i.e., number of days per week, number of minutes per day) participants have spent being physically active (e.g., moderate physical activities, vigorous physical activities, walking) in the last 7 days. Continuous scores are calculated for walking, moderate-intensity, vigorous-intensity, and total activity by multiplying the frequency of activity by the duration of activity for each domain. The 1-item Stanford LCAT [66] is a categorical measure that assesses the type and frequency of physical activities participants have done over the last month. Participants are provided with six descriptive categories of activity ranging from “inactive” to “very active.” The description of each category includes a range of examples of different activities that may fall within that category.

#### Spiritual well-being

The 12-item Functional Assessment of Chronic Illness Therapy-Spiritual Well-being Scale (FACIT-SP) [67] short form assesses participants spiritual wellbeing in the last 7 days. Items are rated from 0 “not at all” to 4 “very much,” and three subscale scores can be calculated to assess meaning, peace, and faith.

#### Psychological flexibility

The 7-item Acceptance and Action Questionnaire-version 2[68] assesses psychological flexibility. Consistent with the cancer-specific Acceptance and Action Questionnaire, participants are provided with a variety of statements related to their cancer experience (e.g., “I am afraid of feelings about my cancer”) and asked to rate how true each statement is for them from 1 “never true” to 7 “always true.” Items are summed, with higher scores indicating greater inflexibility.

#### Values

The 10-item Valuing Questionnaire [69, 70] assesses the extent to which participants worked towards/lived within their personal values in the past-week. Items (e.g., “I felt like I had a purpose in my life”) are rated from 0 “not at all true” to 6 “completely true.” Two subscale scores can be calculated to reflect progress towards values and obstruction of valued action.

## Power analysis

Power analyses were conducted using G-Power [71] to determine the most appropriate sample size for the pilot randomized controlled trial. One-sided binomial tests of *α* = 0.05 were used to calculate power at various sample sizes [72] based on feasibility and acceptability rates found in our prior studies [73]. Sample sizes of 20, 40, and 60 would provide 38%, 70%, and 77% power, respectively, to detect a feasibility rate (i.e., session completion) of 75% or lower if the true feasibility rate in the population is 88%. Sample sizes of 20, 40, and 60 will provide 59%, 84%, and 93% power, respectively, to detect an acceptability rate of 80% or lower if the true acceptability rate in the population is 94%. Aim 3 examines the distributions of change over time for the intervention and control arms. As recommended by Moore et al. [72], the anticipated level of precision of statistical estimators for variables of interest at three different sample sizes (20, 40, and 60) was calculated using longitudinal data from a sample of YA cancer survivors obtained by the research team. As the sample size increased, the precision in our estimates of the mean difference for variables of interest increases. Based on these calculations, the accrual goal was 60 participants (30 per arm).

## Statistical analysis

### Aim 1

Descriptive statistics (mean, standard deviation, percentage, etc.) will be used to examine feasibility, acceptability, and satisfaction data. Feasibility, acceptability, satisfaction, and attrition rates will be compared between groups using Chi-square or Fisher’s exact tests, as appropriate.

### Aim 2

Analyses will be based on intention to treat principles [74]. We will compare the baseline characteristics of participants who are lost to follow-up or drop out with active participants. We will examine incomplete data patterns. We will consider using multiple imputation procedures and other sensitivity analyses as an alternative to intent to treat analyses as needed [75]. Changes from baseline will be estimated for each patient and compared between arms using unadjusted statistical testing. Multilevel longitudinal models will be used to examine the rate and trajectory of change in the intervention targets (i.e., symptom intensity, symptom interference). Modeling approaches will be considered to account for the change from waitlist to intervention for the control arm, including conducting analyses separately for individuals in the control arm during the waitlist period (baseline through A3; *n* = 30) and for all participants during their intervention period (pre- and post-intervention, 3-month follow-up; *n* = 61). Observations will be nested within individuals and individuals will be nested within cohorts. Modeling will be conducted under a mixed effects framework or a generalized estimating equations framework depending on the structure of the data and the missingness pattern. Due to per-patient variation in the time from the baseline assessment to initiation of intervention sessions, time will be considered as chronological (months from baseline), as well as by timepoint (assessment 1, assessment 2, etc.).

### Exploratory aim

Bootstrap mediation will be used to examine the hypothesis that change in self-efficacy and social support from baseline to post-treatment will mediate group differences in outcomes (i.e., symptoms, symptom interference, and health behaviors) over time [76–78]. Bootstrap mediation analyses can be applied when the sample size is small or moderate (e.g., *n* = 20–60) [79, 80].

## Dissemination

Select members of the study team will have access to the final trial data. Final trial results will be published in peer-reviewed journal articles, which will be shared with interested study participants. Upon request, the final dataset, with identifiers removed, may be made available to qualified investigators. Despite being stripped of identifiers prior to release for sharing, there remains the possibility of deductive disclosure of subjects with unique characteristics from the final dataset. Thus, all investigators requesting to use the data will be required to obtain IRB approval and sign a data use agreement before the data will be released.

## Discussion

This study evaluates the feasibility and acceptability of a newly developed group-based behavioral symptom management intervention to improve YA survivors’ abilities to manage physical symptoms (i.e., pain, fatigue) and emotional distress. We chose to target YA survivors within 2 years of completing curative therapy as the symptoms that survivors experience during this time may be long-lasting rather than an acute response to treatment [81, 82]. Additionally, this time period has been referred to as the reentry period during which survivors are beginning to return to “normal” life post-cancer; the reentry period has been identified by many YA cancer survivors as presenting significant psychosocial and symptom management challenges [83, 84]. Thus, YA may particularly benefit from receipt of a behavioral symptom management intervention during this time period.

Though considered, neither survivors’ symptom type nor symptom severity were included as eligibility criteria given the diversity of cancer types experienced and treatments received by survivors in our target population. Rather, the intervention provides YA with a menu of coping skills that can be used to address both persistent (e.g., fatigue) and episodic (e.g., anxiety prior to scans) symptoms. Once deemed feasible and acceptable, future studies evaluating the efficacy of the intervention may benefit from specifically targeting YA survivors experiencing clinically significant levels of pain, fatigue, and/or distress.

The intervention, developed with input from YA cancer survivors and oncology providers, is a novel combination of traditional behavioral symptom coping strategies, home-based physical activity, strategies from contemporary cognitive-behavioral theory-based approaches like ACT [34] and those to foster self-compassion, concepts from MCP [38–41], and strategies to improve communication and healthcare engagement. The intervention has several key features. It was designed to meet the unique concerns of this patient population through a novel combination of skills from multiple complementary evidence-based treatment approaches (e.g., behavioral symptom coping skills, ACT, MCP). To enhance social connectedness, provide YA cancer survivors the opportunity to interact with same-aged peers who have been through similar experiences, and reduce feelings of isolation, this intervention is offered in a group format. Our work [33] and the work of others [53, 54, 85] suggest that YA have limited opportunities to connect with other YA with cancer following diagnosis and often feel as though their peers, family, and members of their community do not understand what it is like to be a young person who has had cancer. The group format allows participants to connect with others who understand and relate to their experiences.

The intervention was also designed to be accessible for YA cancer survivors who are often juggling other competing life demands, including their careers and families [14]. The intervention is delivered remotely using videoconferencing technology, on days and at times that are individually determined to be feasible for each cohort (e.g., early mornings, weekday evenings, weekends). Individual make-up sessions are offered to participants who are unable to attend a group session allowing participants to more easily return to the group sessions when next able.

The intervention provides participants with a study-specific mobile application, which accompanies the face-to-face intervention sessions. The National Institutes of Health [86] have stressed the importance of developing interventions that balance technology with “human touch.” While the development of hybrid interventions has been on the rise, and these interventions have been successfully used to deliver behavioral symptom management strategies [87, 88], to the best of our knowledge, ours is one of the first to target YA cancer survivors. The inclusion of mobile health technology to support home practice of intervention skills as well as on-the-go use of content is particularly relevant to YA. YA are among the most technologically connected group in the USA, and mobile application use is highest among this population [89, 90].

There are several additional strengths. First, the study design allows the waitlist arm participants to serve as a comparison group for participants in the intervention arm. This allows us to examine differences in patterns of change in outcomes between the intervention and control arms while also providing a larger sample to examine feasibility and acceptability data. Second, given the paucity of behavioral symptom management interventions targeting the unique needs of YA cancer survivors, this study design allows for all study participants to receive access to a potentially beneficial intervention. Third, patient-reported outcome measures selected for use in the study have strong reliability and validity data and map directly on to intervention targets and proposed treatment effects.

In addition to the strengths noted, there are a few notable limitations. This study is being conducted within one National Cancer Institute-designated comprehensive cancer center in the southeastern US. Therefore, results from this study may not generalize to the experiences of patients living in other regions or treated in community settings. This study also recruited YA cancer survivors with specific cancer types and those who have completed cancer treatment and have no evidence of disease. Thus, the results may not generalize to cancer survivors on active treatment, those with metastatic disease, and to those who were diagnosed with cancer types not captured within this study. Furthermore, participants recruited were primarily white (72.1%) and non-Hispanic (91.8%), which may further limit generalizability to other racial and ethnic groups. While control arm participants receive the behavioral symptom management intervention after completing the third assessment, these participants are receiving usual medical care prior to starting the intervention. Future randomized controlled trials examining the efficacy of this intervention would benefit from including an active control condition that accounts for time, attention, and support.

Of those screened for eligibility, 40.5% consented to participate. This number is slightly higher, though similar, to recruitment metrics for other behavioral interventions targeting YA cancer survivors [91, 92] and studies involving patient-reported outcomes [93] YA’s reasons for declining participation pointed to the significant competing demands experienced by this population (i.e., 53.3% of those who declined said they were too busy) and suggest the need for increased flexibility in intervention delivery and the value of communicating about flexible components of the intervention (e.g., ability to attend make-up sessions) at the time of recruitment. An additional 38.6% of those who declined were not interested in the study or not interested in participating in any research. YA cancer survivors are one of the least represented groups in clinical research [94, 95]. Education about the value of participation in clinical research for YA cancer survivors is warranted.

The results from this study have the potential to significantly impact future research and ultimately, the care of YA cancer survivors. If this study is found to be feasible and the intervention acceptable, larger randomized controlled trials may be conducted to further examine the efficacy of the intervention and its impact on important outcomes (e.g., pain severity/interference, fatigue, emotional distress, and symptom interference more broadly) for YA cancer survivors, an understudied population for whom high physical and emotional symptom burden may be particularly impactful. In addition, results may positively impact the care of YA cancer survivors. The developed intervention could be offered to YA cancer survivors through programmatic initiatives in the formal Teen and Young Adult Oncology Program at this institution, and potentially more broadly due to the flexibility of the videoconferencing-based intervention delivery and use of the mobile application.

### Supplementary Information

Supplementary Material 1.

## Data Availability

Not applicable.

## Abbreviations

YA: Young adult
U.S.: United States
S.M.A.R.T.: Specific, measurable, achievable, relevant, time-oriented
PMR: Progressive muscle relaxation
A1: Assessment one
A2: Assessment two
A3: Assessment three
A4: Assessment four
A5: Assessment five
TAQ: Treatment Acceptability Questionnaire
STTS-R: Satisfaction with Therapy and Therapist Scale-Revised
PROMIS: Patient Reported Outcomes Measurement Information System
IRB: Institutional Review Board
REDCap: Research Electronic Data Capture
R: Randomized
Mos: Months
app: Application
